# What the Cat Dragged in: Quantifying Prey Return Rates of Pet Cats (*Felis catus*) With Outdoor Access in the UK

**DOI:** 10.1002/ece3.71063

**Published:** 2025-03-06

**Authors:** H. L. Lockwood, M. Bulling, M. Huck

**Affiliations:** ^1^ College of Science and Engineering University of Derby Derby UK

**Keywords:** conservation, detectability, domestic, free‐roaming cats, Great Britain, palatability, predation, wildlife

## Abstract

Non‐native predators can cause great harm to natural ecosystems through competition for resources and by directly predating on native species. Domestic cats ( *Felis catus* ) predate on wild prey throughout the world and have been implicated in a number of species declines. However, in the UK, long‐term, widespread research is lacking. Here, the study aimed (i) to quantify prey returned home across the country and (ii) to investigate factors which may influence these return rates. A predation survey was conducted on 553 cats across the UK for up to 43 months (2018–2021), recording all prey returned home and subsequently detected by the cats' owners. All owners of cats with outdoor access were encouraged to participate, the only exclusion criterion being indoor‐only. Data were gathered upon registration regarding the age, sex, and body condition of participating cats, allowing for the analysis of the potential influence of such factors. It was estimated here that the current UK population of pet cats (10.8 million total) return a total of between 37.25 million and 140.4 million prey per year, the majority being mammals (83% of detected prey). Sex, age, and body condition of cats, along with the presence of a cat flap, whether a bell was worn, level of urbanisation, and the season of data collection all had a statistically important effect on prey return rates. While most cats returned 0–1 prey per month, a small minority (*n* = 3 cats) returned over 15 individuals monthly. It is important that true predation rates (in addition to the return rates found here) are further explored and quantified, along with the actual impact that this has or does not have on prey populations. Future efforts to limit the impact of cat predation should focus in particular on identifying super predators with a view to limiting their predation.

## Graphical abstract

This study used predation survey techniques (citizen science) in order to quantify prey return rates across the UK, along with potential influencing factors. Although between 37 and 140 million prey were estimated to be returned home by cats, it is important to recognise that this does not indicate impact on prey populations. Therefore, further research is necessary into the ecological relationships between cats and their prey.

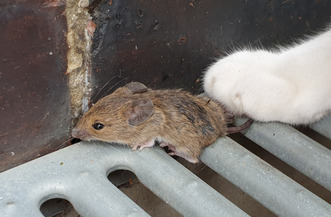

## 1 Introduction

Domestic cats, both feral and owned, can have a detrimental effect on native wildlife populations around the world. This can be a result of disease transmission (Gerhold and Jessup 2013) and ‘fear effects’ (Beckerman et al. 2007; Bonnington et al. 2013) due to their presence, but prey populations can also be directly affected by cats' predatory habits (Doherty et al. 2016).

Cats are formidable hunters of rodents in particular, as these species commonly live in burrows, and a cat can lie in wait in a ‘watch’ posture for a long time (Turner and Bateson 2000). Upon the prey's emergence, the cat is able to propel itself towards its prey horizontally, close to the ground (Leyhausen 1979). Hunting birds is also possible when they are on the ground (and occasionally perched higher up), and cats can have a particular impact on ground‐nesting birds in certain locations where such species are prevalent (Oppel et al. 2014). Birds are more likely than mammals to avoid capture since they are able to escape vertically where cats cannot follow (Leyhausen 1979). In the majority of dietary studies on cats, mammals are the most frequently recorded taxon worldwide (constituting a mean of 64.4% of prey in return surveys and 70.8% in scat and stomach analyses, see Lockwood 2024 in review for a full evaluation). This is followed by birds, constituting means of 24.8% of return survey data and 16.9% of scat and stomach study data (Lockwood 2024). Herptiles are globally the least often returned (questionnaire) and eaten prey (scat and stomach studies), at a mean of 10.8% and 11.6%, respectively (Lockwood 2024). The condition of returned prey (whether whole or part‐eaten, for example) can be used to infer the palatability of different taxa. For example, shrew species are commonly returned whole, whereas mice are frequently eaten (Toner 1956; Leyhausen 1979; Turner and Bateson 2000; Krauze‐Gryz et al. 2012; Kauhala et al. 2015). This may suggest that mice are more palatable to cats than shrews. However, prey and quantities caught may also depend on the type of cat (whether feral or owned pets).

There is a large difference in the number of prey caught by feral and owned cats (Krauze‐Gryz et al. 2012), with feral individuals being reliant on wild prey. In countries containing vast rural areas, such as Australia, cat populations are often largely made up of feral individuals, killing a far greater number of wild prey animals per individual cat than their owned counterparts (Bonnaud et al. 2007). In densely populated and comparatively urbanised countries, such as the UK, there are relatively few feral cats. Recent estimates suggest that there are around 281 people in the UK per km^2^ (Office for National Statistics 2024), compared with around 35 people per km^2^ in the USA (US Census Bureau 2024) and only 3 per km^2^ in Australia (Australian Bureau of Statistics 2024). In the UK, the majority of cats are pets, being fed and housed by people in a domestic setting (PDSA 2024). Of these owned cats, between 69% and 73.9% have access to outdoor space, unsupervised (Finka et al. 2019; PDSA 2024), with the remaining individuals being confined to indoor spaces. Most prey species in the UK have evolved with a similar predation pressure from either European wildcats ( *Felis silvestris* ) or other terrestrial predators such as mustelids (Badenes‐Pérez 2023). Although this has led to escape and avoidance behaviours developing in some prey species, pet cats exist at unnaturally high densities (in the UK, a mean of 331 cats per km^2^ from three studies, Baker et al. 2005; Baker et al. 2008; Sims et al. 2008), compared with European wildcats and hybrids in Scotland (0.7 individuals per km^2^, Kilshaw et al. 2015). Therefore, depending on how frequently they hunt, pet cats may have a negative impact on British prey species.

The degree to which each individual pet cat hunts can vary considerably. The majority of pet cats are reported to take (or at least return) few or no prey at all (Churcher and Lawton 1987; Morgan et al. 2009; van Heezik et al. 2010), whereas a number of ‘super predators’ are found to increase the mean, often far surpassing the median (van Heezik et al. 2010). There is often large variability between individual cats' return rates (Barratt 1998; Morgan et al. 2009; van Heezik et al. 2010; Kauhala et al. 2015). Kauhala et al. (2015), for example, found that 9% of the pet cats studied returned 40% of the total prey recorded, and Morgan et al. (2009) state that 80% of pet cats in the study returned 10 or fewer prey over the whole year. However, it is important to note that not all captured prey animals are returned home. Indeed, the proportion of caught prey being returned home by cats has been estimated to range from 8.8% (Krauze‐Gryz et al. 2012) to 50% (George 1974).

There are many factors that may affect a cat's propensity to hunt wild prey. Some are controllable such as attaching a belled collar or bib to the cat (Calver et al. 2007; Gordon et al. 2010). Belled collars have been found to reduce total prey returned by 53% (but prey returned does not equal total capture rates, Gordon et al. 2010), although this measure is thought to primarily reduce mammal mortality while having little effect on bird kills (Woods et al. 2003). In addition, it has been found that neutered cats (both male and female) return prey more frequently than entire individuals, although it should be noted that samples were highly unbalanced with 95%–98% being neutered (Barratt 1998; Kauhala et al. 2015). Another measure may be the absence of a cat flap, since cats' outdoor activity can be limited more easily at particular times of day where there is no cat flap installed. Additionally, the presence of a cat flap may increase the proportion of prey captures that are then returned and detected by owners, since cats have the freedom to bring their prey indoors. Recently, toys and play interaction have been found to both increase (Panchana et al. 2025) and reduce (Cecchetti et al. 2021) predation behaviours in pet cats, suggesting that play may alleviate the desire to hunt in some cats but hone the predation abilities of others.

Intrinsic factors which can affect predation rates include the cat's age and body condition, as age has been found to be negatively correlated with the number of invertebrates, birds, and lizards returned (Gillies and Clout 2003), and those of overweight condition (with a higher Body Condition Score, BCS) may return fewer birds than slimmer individuals (Woods et al. 2003). When studying cats in both urban and rural locations in Australia, McGreevy et al. (2008) found a link between urbanisation and BCS, with obesity being more prevalent in rural areas. This is in contrast to other studies finding no significant association between the two variables (in France, Colliard et al. 2009; and Australia, Murphy et al. 2023). Differences in the predated taxa between urban and rural environments have also been observed. For example, urban pet cats have been found to kill three times as many birds (per cat) as rural individuals (also pets) in Poland, suggesting increased bird densities and availability in urban areas (Krauze‐Gryz et al. 2017), with a similar pattern being observed in owned cats in both Australia (Barratt 1997) and Finland (Kauhala et al. 2015). In contrast, over twelve times as many shrews were returned by rural pet cats (3.8 per cat) than those in urban environments (0.3 per cat) in the same Polish study (over a mean of 12 months per cat, Krauze‐Gryz et al. 2017). Proximity to and accessibility of certain habitats is important when assessing the overall impact of predation by cats, as native, protected, and vulnerable prey species may be more easily negatively affected by cats living close to, for example, nature reserves (Kays and DeWan 2004; Seymour et al. 2020). The overall impact of predation by cats depends rather heavily on location and local faunal composition (Badenes‐Pérez 2023).

While sex has frequently been found to have no significant effect on prey captures or returns (Morgan et al. 2009; van Heezik et al. 2010; Tschanz et al. 2011; Loyd et al. 2013; Kauhala et al. 2015; McDonald et al. 2015), it is important that it is considered in all behavioural research. Other uncontrollable variables may include the time of year (season) and the year itself, with some prey species populations experiencing boom and bust cycles (Merritt 2010; Andreassen et al. 2021), thus affecting their availability. The boom and bust cycles of prey are not often observed in dietary studies, as this requires a multi‐year design.

Due to seasonal differences in prey availability, it is important to record data over a full 12‐month period. Currently, there are few British studies that monitor cat predation on a large scale and over an adequate period of time. Most research in the UK has focussed on a particular city, town, or village (e.g., Churcher and Lawton 1987; Baker et al. 2005, 2008; Thomas et al. 2012; Pirie et al. 2022). While studies such as Woods et al. (2003) monitored a large cat population, returns were only recorded over two seasons. Many previous studies have been based on postal surveys (e.g., Woods et al. 2003; Thomas et al. 2012) or telephone surveys (Robertson 1998), but since the majority of UK residents now have easy access to online devices, as well as technological competency, an internet‐based study using email, digital photographs, and spreadsheets is likely to produce a large response. Indeed, some previous studies have used digital means, such as advertising via social media platforms, to great effect (Mori et al. 2019; Mella‐Méndez et al. 2022; Panchana et al. 2025). In addition, digital data can be easier and more secure for researchers to store and work with than paper documentation. Here, a UK‐wide, long‐term online survey was conducted in order (i) to quantify the average number (per cat) of vertebrate prey returned home by cats in the UK and (ii) to examine the factors that may influence such predation rates. It was hypothesised that the cats in this study would predate mainly on mammals, due to their wide availability and cats' physical adaptations to capturing them (Leyhausen 1979). Since many studies have reported no effect of sex, it was hypothesised that this would not influence predatory behaviour (Barratt 1998; Gillies and Clout 2003; Loyd et al. 2013; Kauhala et al. 2015). It was expected that younger cats (Gillies and Clout 2003) and those of a slimmer body condition would return a greater number of prey (Woods et al. 2003). In addition, given a balanced dataset, it was hypothesised that cats that were neutered would have a lower return rate than those that were entire, following previous research (Robertson 1998).

Those wearing a belled collar were hypothesised to return fewer prey than those without, which may be particularly apparent for avian prey (Calver et al. 2007; Gordon et al. 2010). It was also hypothesised that those with access to the home via a cat flap would yield a greater number of records, since prey may be returned indoors, increasing detectability. As the season has previously been found to influence the numbers of prey returned home by cats, with increased rates in the spring and summer when prey are more active and available (Thomas et al. 2012), the same was hypothesised here. This was also hypothesised to vary by taxon, since Krauze‐Gryz et al. (2017) found that while shrews and birds returned by cats peaked in June, rodents were most frequently recorded in September, and reptiles in April. In addition, return rates were predicted to vary by year (as this is a multi‐year study), potentially following the boom‐bust cycles of small mammal species. However, as such long‐term studies are rare, this has not previously been monitored. Taxa returned can also depend on how urban or rural the local environment is and the prey availability of those areas. It was, therefore, hypothesised that a greater proportion of prey returns in urban areas would be birds than in more rural locations (Krauze‐Gryz et al. 2017).

The majority of prey returned were expected to be dead, following previous studies (Baker et al. 2005; Thomas et al. 2012) and most brown rat ( *Rattus norvegicus* ) and rabbit ( *Oryctolagus cuniculus* ) returns were likely to be juveniles (Fitzgerald et al. 1991; Flux 2007). Since insectivores appear to be unpalatable (Leyhausen 1979; Krauze‐Gryz et al. 2012; Kauhala et al. 2015), it was hypothesised that most shrews (*Sorex* spp.) would be returned whole and intact.

## 2 Materials and Methods

### 2.1 Data Collection

In order to attract a large sample of cat owners from across the UK, a website was produced and marketed through online social media channels (Facebook and Twitter), email mailing lists, radio interviews (17 local radio stations), and magazine advertisements (e.g., New Scientist, Liverpool 2020). Registration was open to new cats from May 2018 to May 2021, and data were collected from June 2018 until December 2021. The data presented here also include two cats for which prey return data were collected from 2014 and 2017. Any cat owner residing in the UK (Great Britain and Northern Ireland), whose cat had access to the outdoors, was encouraged to sign up.

Cats were first registered online using a questionnaire (see S1 for all questions). This questionnaire asked a variety of questions about the cat, including age, sex, body condition (using imagery, see S2), and whether a belled collar was worn. These questions were compiled using findings of previous studies in the UK (Woods et al. 2003; Baker et al. 2008; Thomas et al. 2012; McDonald et al. 2015) and elsewhere in the World (Robertson 1998; Flux 2007; Gordon et al. 2010; Tschanz et al. 2011).

A joining pack was sent to each cat owner via email, including a Microsoft Excel ‘cat diary’ document and help guide. Participants were provided with a mammal identification guide in the form of a PDF and URL, along with a link to the Royal Society for the Protection of Birds (RSPB) bird identification website (RSPB 2022) and herptile identification PDFs available from the Amphibian and Reptile Groups UK (ARG UK 2017). This helped to improve the accuracy of identification and was of particular importance where photographs were not submitted to accompany prey records.

Each month (for a minimum of 12 months from the joining date), participants were emailed to request data submission through an online portal system. This required cat owners to fill in details of their cat's prey returns, including species identification, date and time of detection, whether the animal was returned whole and intact, had been partly consumed, or observed to be fully eaten, and any additional notes (including, for example, age or life stage of prey). Where identification to species level was not possible, or owners were not confident in their identification, they were asked to categorise prey (e.g., as ‘small mammal’, ‘mouse’ or ‘bird’). Participants were also asked to take a photograph of each prey return (where possible) and send these along with the cat diary to confirm species identification and age of the individual, where appropriate. Photographs were taken for almost half of the prey returned (46.37%).

### 2.2 Data Preparation

Where a prey item was returned to a multiple‐cat household and the owner could not say with any certainty which cat returned the item, the prey was randomly assigned to one individual (based on a random number generator). This practice was necessary for 3.6% of all data month values.

From the recorded prey return dates, data were categorised by season: winter (December, January, February), spring (March, April, May), summer (June, July, August), and autumn (September, October, November), according to the meteorological calendar (Met Office 2024). The year was altered so that each season contained months of the same data collection year. For example, where December 2019, January 2020, and February 2020 formed one winter season, December's year was altered to 2020. Only full years were included in the analysis (2019, 2020, and 2021). The age of cats was recorded in three‐year categories (plus the ‘kitten’ age group of < 1 year) at the time of registration. This was then further categorised into four groups: ‘kitten’ (< 1 year), ‘young adult’ (1–6 years), ‘mature adult’ (7–12 years), and ‘senior’ (≥ 13 years). Age was also altered where individuals had been registered with the project for more than 2 years (it is assumed that their age increased by one three‐year category after two full years of data recording).

Each owner's postcode was converted into approximate coordinates using an online converter (Grid Reference Finder 2024). These points were then mapped using GIS software (QGIS Development Team 2023), where spatial analytical tools were used to calculate the proportion of built‐up habitat within each cat's buffer zone. Buffers were based on maximum ranging distances found by Thomas et al. (2012), specifically the radius of the absolute maximum daily range (188 m). The percentage of each buffer made up of built‐up habitat was recorded as a continuous variable. ‘Built‐up’ areas were those classified as such by Department for Environment Food and Rural Affairs (2024) in England and were classified using satellite images (Google 2024) for Scotland and Wales (none of the cats in this study resided in Northern Ireland). Land classified as built‐up was primarily made up of buildings and gardens, as well as car parks and industrial land (Department for Environment Food and Rural Affairs 2024). The area covered (Great Britain and Northern Ireland) contains varying landscapes, including mountainous areas, arable land and pasture, wetlands, woodlands, and urbanised areas. The latitude of cat locations ranged from 50.1° N to 59.3° N, and the average temperature for the whole area across the study period was 10.9°C per month, ranging from 3.3°C to 18.7°C (Statista 2025).

### 2.3 Formal Analyses

Due to a very large number of zeros in the data and the overall very low return rates, reptiles and amphibians were not included in the formal analyses. Similarly, invertebrates were occasionally recorded but not consistently, and they are therefore excluded from analyses.

Generalised Variance Inflation Factors (GVIFs) were first generated in a frequentist framework, along with the standardised GVIFs (GVIF^(1/(2*df))). All of these values were below four and, therefore, no multicollinearity was evident (Fox and Monette 1992). Bayesian Generalised Linear Mixed Models (GLMMs) were then used to analyse the effects of each variable on the number of mammals and birds returned home by cats in a month. Model selection involved comparing four different error structures to determine which was most suitable. These models were: Poisson, zero‐inflated Poisson, zero‐altered Poisson (hurdle model), and a negative binomial model. After comparison, the Poisson model was found to be overdispersed and thus the data did not follow a Poisson distribution. The hurdle model assumes that there are no false zeroes, but as the detectability of returned prey may vary due to taxon or the location of return (indoors or outdoors), this was also unsuitable. On comparing the zero‐inflated Poisson and negative binomial models and conducting out‐of‐sample predictions (where 10% of data are dropped and then predicted) for each, the negative binomial approach was found to be most suited to the dataset and was therefore used here. The final model used 20,000 iterations with a burn‐in of 8000 iterations (thinning rate of five and three chains). Hence, the total number of iterations used was 3 × (20,000—8000)/5 = 7200 iterations. Chain convergence (or mixing) was assessed at each stage by plotting each chain against its iteration number (as outlined by Zuur and Ieno 2016). Models were updated to include further iterations or reduce burn‐in until mixing was visually sufficient (trace plots available in S3).

Due to the length of time taken for each of these models to run and the computer memory requirements, statistically important variables (the terminology used in Bayesian statistics) were identified in two stages. This involved assessing the suitability of one half of the variables at a time. The first set included the following variables: sex (male, female), age (kitten, young adult, mature adult, senior), whether neutered, and whether a cat flap was accessible. The second set of variables tested was: body condition (A, B, C, D), whether a bell was worn, season (winter, spring, summer, autumn), year (2019, 2020, 2021), and the percentage of built‐up land within a 188 m radius of home. Those variables that were found to be statistically important were selected for inclusion in the final model. The selection of each subset of variables grouped in this process was random, and this was conducted again with a different selection, having no impact on the final variable selection. All variables were tested with and without a possible interaction with taxon.

All GLMM analyses were conducted in a Bayesian framework, as the R packages available for zero‐inflated mixed models in a frequentist context are very limited. Analyses were completed using R version 4.0.2 and R Studio version 1.0.153 (R Core Team 2016; R Studio Team 2020), utilising the packages ‘R2jags’ (Su and Yajima 2020) and ‘cars’ (Fox and Weisberg 2019), along with BUGS code from Zuur and Ieno (2016).

## 3 Results

**FIGURE 1:**
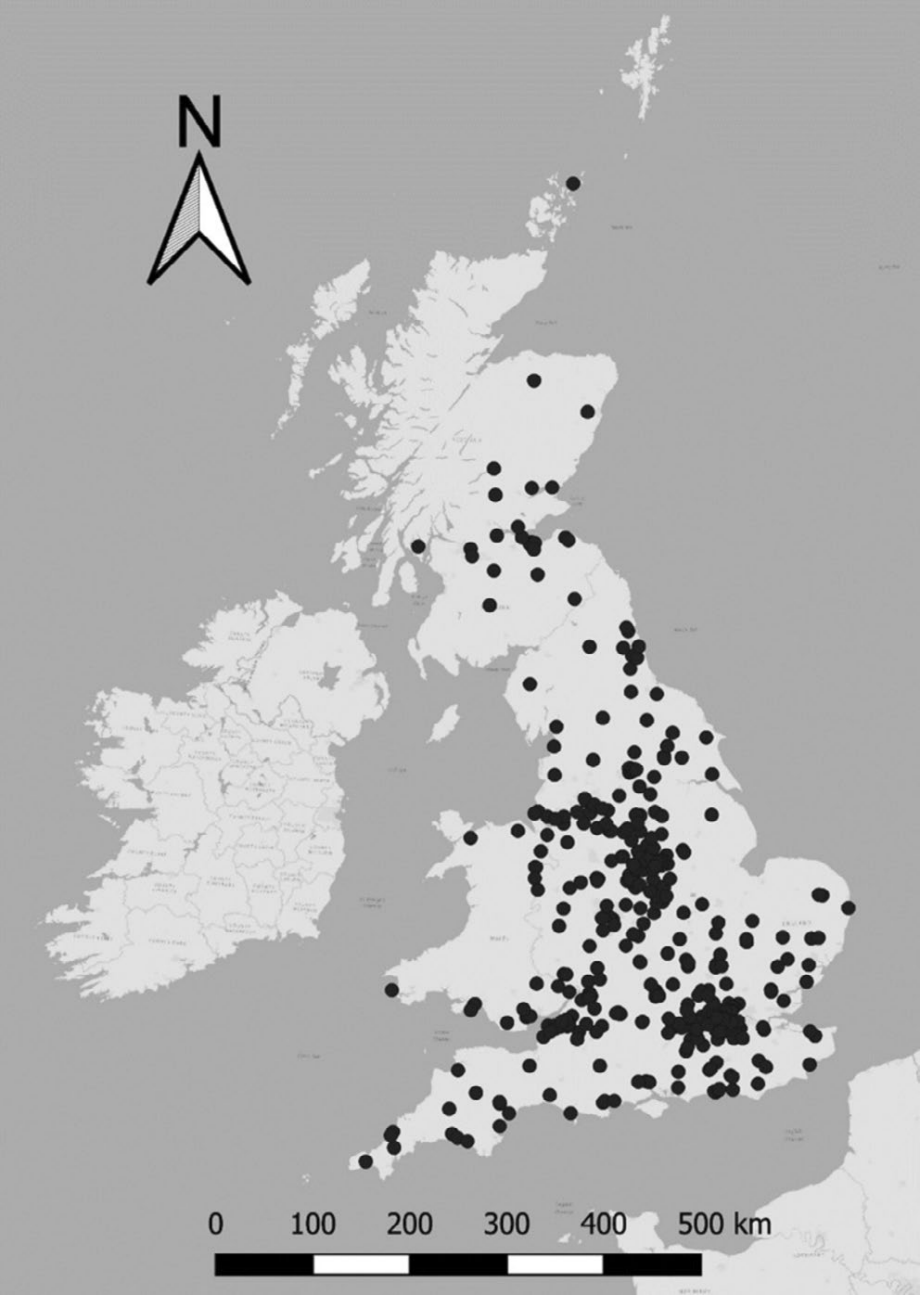
Locations of 553 cats in the UK, for which prey return data were collected.

Predation data were collected for 553 cats located across the UK, with higher densities of respondents in areas of larger human population (around London for example, Figure 1). The number of data months for each cat ranged from only one month to 43 months, giving a total of 4674 data months for all 553 cats. While for the majority of these months, zero prey were recorded (2987 months = 64%), prey returns ranged up to 61 in a single month for one cat. All prey species records can be found in S4. Based on owner estimates, cats spent a mean of 9.6 h out of the house per day in the warmer months (median = 9.25 h) and 5.7 h out of the house per day in the colder months (median = 5 h), although alternative shelter may be sought during these time periods.

A total of 7013 prey were returned by 553 cats across a 4674 data month period, producing a mean monthly estimate of 1.5 prey per month (median = 0) or 18 per year, per cat with outdoor access in the UK. However, most cats (69.08%) returned a mean of ≤ 0.3 prey per month (≤ 3.6 prey returned annually). While mean monthly values varied (between 0.59 and 3.75 per cat), most monthly medians (for all cats) were zero (with just four exceptions out of 43 project months). These higher medians appear to be largely due to a lower sample size for some months (minimum *n* = 16) compared to others (maximum *n* = 218), as well as season. Where medians are zero, most cats returned no prey for that given month. Although most cats returned a mean of between zero and 0.99 prey per month, a very small number (*n* = 8) were found to return a mean of 10 prey items or over each month, including three returning more than 15 prey per month (S5). Here, these cats are termed ‘super predators’.

Using the mean value of 18 prey per year, along with an estimated 7.45 million outdoor‐going cats in the UK (PDSA 2024), it can be estimated that around 140.4 million prey are returned across the country annually. Here, the median per month was zero, and thus could not be extrapolated. However, using a subset of 157 cats submitting 12 consecutive months of data, a median of 5 prey (per year) was produced (mean = 15.2). This led to an estimate of 37.25 million prey returned per year by British cats (again, derived from 7.45 million outdoor‐going cats, PDSA 2024).

Most prey species returned home in this study were mammals (83.21%), followed by birds (16.03%), and a comparatively small number of amphibians (0.61%) and reptiles (0.14%). Per cat (per month), this equates to 1.31 mammals, 0.25 birds, 0.009 amphibians, and 0.002 reptiles. Photographs were received for 46.37% of all prey species.

### 3.1 Influencing Factors

In order to determine the factors which may influence predation, the final model described here included (apart from taxon) eight factors: sex, age, body condition score, whether a cat flap was installed, season, year, whether a belled collar was worn, and level of urbanisation, some of which showed an interaction effect with taxon. The neutering status of cats was not included in the final model, as no statistically important (terminology used concerning Bayesian analyses) effect was indicated by the initial process of model selection (S6). In addition, the neutering status was greatly unbalanced, with 97.8% of cats (*n* = 541) being neutered. The categories and sample sizes are given in Table 1 and S7, respectively.

**TABLE 1:** Details of each categorical variable used in the final model (as outlined in Table 2).

Variable	Category	Description
Taxon	Mammals	Mammalian prey
	Birds	Bird prey
Sex	Male	Male cat
	Female	Female cat
Age	Kitten	< 1 year
	Young adult	1–6 years
	Mature adult	7–12 years
	Senior	13+ years
Body condition	A	BCS 1–3
	B	BCS 4–5
	C	BCS 6–7
	D	BCS 8–9
Cat flap	Yes	Access to catflap
	No	No access to catflap
Bell	Yes	Belled collar worn
	No	No belled collar worn
Season	Winter	December–February
	Spring	March–May
	Summer	June–August
	Autumn	September–November
Year	2019	Full year 2019
	2020	Full year 2020
	2021	Full year 2021

**FIGURE 2:**
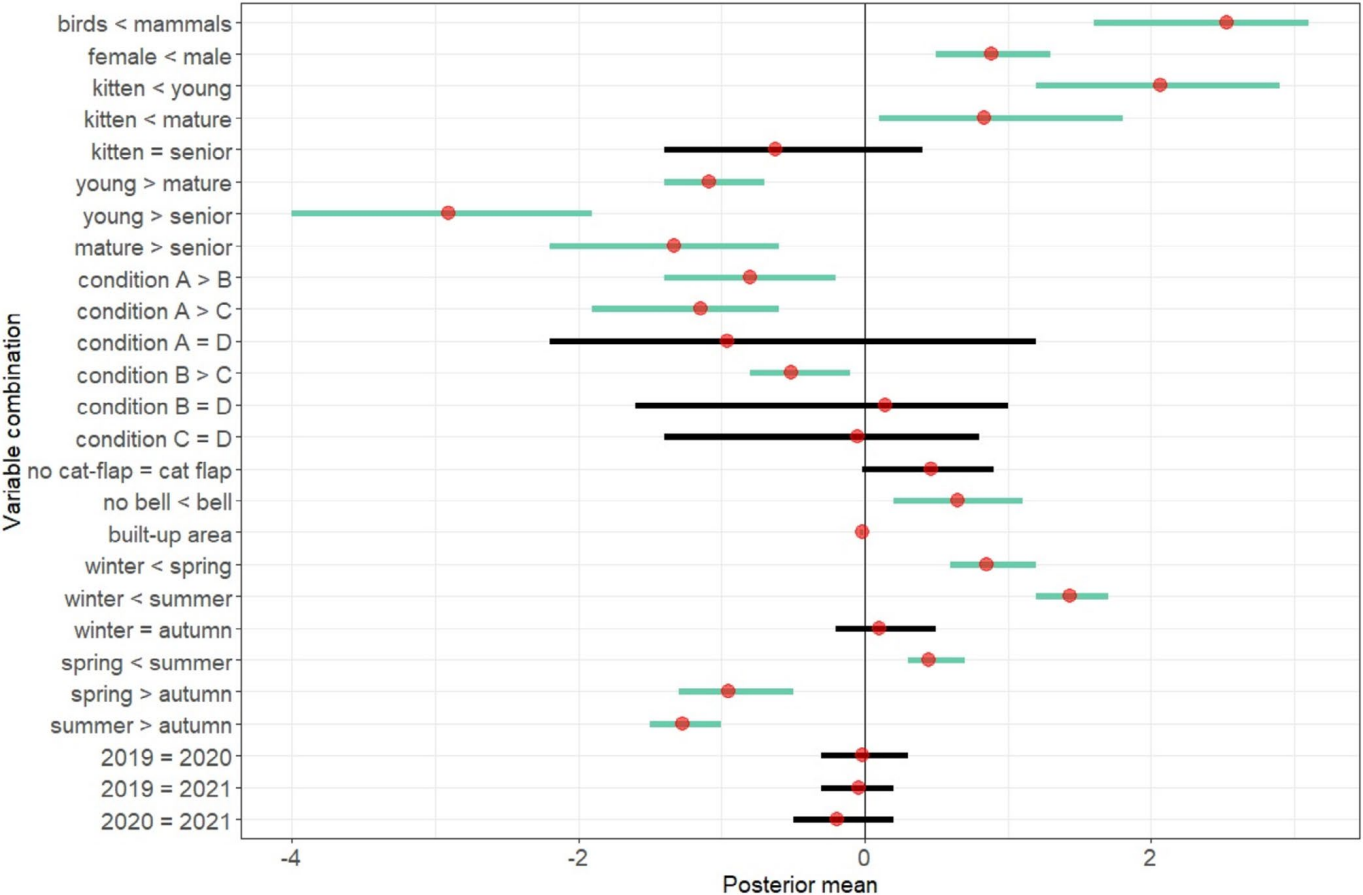
The posterior mean (red points) and credible intervals (horizontal lines) for each variable combination. Where credible intervals cross zero, there is no ‘statistically important’ effect (black lines). Green lines indicate important results, and the direction of the effect is shown on the y axis (<, >, =).

There was an important difference between males and females in the number of prey returned home, with males returning a greater amount (Table 2, Figure 2). However, there was no discernible interaction with taxon here. When comparing young adult cats (1–6 years) with kittens (< 1 year) and mature adult cats (7–12 years), a difference was found between taxa, with birds being less affected by age than mammals. Overall, for age, the numeric outputs show a difference between almost all groups (Table 2).

**TABLE 2:** Numerical outputs of Bayesian negative binomial GLMM, giving posterior mean (P mean), standard error (SE), and the 2.5% and 97.5% credible intervals. Where credible intervals do not cross zero, a statistically important result is observed (given in bold). For body condition categories, see S2.

Variable	*p* mean	SE	2.5%	97.5%
(Intercept)	−4.15	0.4	−4.9	−3.6
Taxon: Birds < Mammals	**2.53**	**0.4**	**1.6**	**3.1**
Sex: Female < Male	**0.88**	**0.2**	**0.5**	**1.3**
Age: Kitten < Young adult	**2.06**	**0.5**	**1.2**	**2.9**
Kitten < Mature adult	**0.83**	**0.6**	**0.1**	**1.8**
Kitten = Senior	−0.62	0.5	−1.4	0.4
Young adult > Mature adult	**−1.09**	**0.2**	**−1.4**	**−0.7**
Young adult > Senior	**−2.91**	**0.8**	**−4.0**	**−1.9**
Mature adult > Senior	**−1.33**	**0.4**	**−2.2**	**−0.6**
Body condition: A>B	**−0.80**	**0.3**	**−1.4**	**−0.2**
A>C	**−1.15**	**0.3**	**−1.9**	**−0.6**
A = D	−0.96	0.9	−2.2	1.2
B>C	**−0.51**	**0.2**	**−0.8**	**−0.1**
B = D	0.14	0.7	−1.6	1.0
C = D	−0.05	0.7	−1.4	0.8
Cat flap: No = Yes	0.46	0.2	−0.02	0.9
Bell: No < Yes	**0.65**	**0.3**	**0.2**	**1.1**
Percentage of built‐up in buffer	**−0.02**	**0.003**	**−0.03**	**−0.01**
Season: Winter < Spring	**0.85**	**0.1**	**0.6**	**1.2**
Winter < Summer	**1.43**	**0.1**	**1.2**	**1.7**
Winter = Autumn	0.10	0.2	−0.2	0.5
Spring < Summer	**0.45**	**0.1**	**0.3**	**0.7**
Spring > Autumn	**−0.95**	**0.2**	**−1.3**	**−0.5**
Summer > Autumn	**−1.27**	**0.1**	**−1.5**	**−1.0**
Year: 2019 = 2020	−0.02	0.1	−0.3	0.3
2019 = 2021	−0.04	0.1	−0.3	0.2
2020 = 2021	−0.19	0.2	−0.5	0.2
Taxon * Sex: Female—Male	−0.17	0.1	−0.4	0.04
Taxon * Age: Kitten—Young adult	**−0.83**	**0.3**	**−1.3**	**−0.4**
Kitten—Mature adult	−0.45	0.3	−1.0	0.3
Kitten—Senior	−0.46	0.3	−1.0	0.1
Young adult—Mature adult	**0.41**	**0.1**	**0.2**	**0.6**
Young adult—Senior	1.07	0.7	−0.2	2.2
Mature adult—Senior	−0.16	0.3	−0.7	0.5
Taxon * Body condition: A—B	0.26	0.2	−0.01	0.6
A—C	**0.37**	**0.21**	**0.1**	**0.7**
A—D	−0.32	0.5	−1.1	0.8
B—C	0.24	0.2	−0.1	0.6
B—D	−0.20	0.5	−1.1	0.5
C—D	−0.81	0.4	−1.7	0.1
Taxon * Cat flap: No—Yes	**0.34**	**0.1**	**0.1**	**0.6**
Taxon * Bell: No—Yes	**−0.86**	**0.1**	**−1.1**	**−0.6**
Taxon * Percentage of built‐up in buffer	−0.01	0.001	−0.01	0.01
Taxon * Season: Winter—Spring	−0.15	0.1	−0.5	0.1
Winter—Summer	**−0.47**	**0.1**	**−0.7**	**−0.3**
Winter—Autumn	**0.43**	**0.1**	**0.1**	**0.7**
Spring—Summer	−0.12	0.2	−0.5	0.1
Spring—Autumn	**0.85**	**0.2**	**0.3**	**1.2**
Summer—Autumn	**0.82**	**0.1**	**0.6**	**1.1**
Taxon * Year: 2019–2020	**0.49**	**0.1**	**0.2**	**0.7**
2019–2021	**0.25**	**0.1**	**0.03**	**0.5**
2020–2021	−0.06	0.2	−0.5	0.3

Cats of body condition ‘A' (BCS of 1–3, see S2 for illustration and categories) were found to return a greater quantity of prey than categories B (BCS of 4–5) and C (BCS of 6–7). An interaction was found between taxon and body conditions A –C, with condition having a greater effect on mammal returns than that of birds. Having a cat flap installed had no statistically important effect on prey returns, although the credible intervals only marginally crossed zero, and the posterior mean of 0.46 rather indicated more returns when a cat flap was present (Table 2, Figure 2). Those cats which wore a belled collar (*n* = 147, 26.6% of cats monitored here) returned a greater number of prey than those without bells, a result which was more strongly observed in birds than in mammals (Table 2).

**FIGURE 3:**
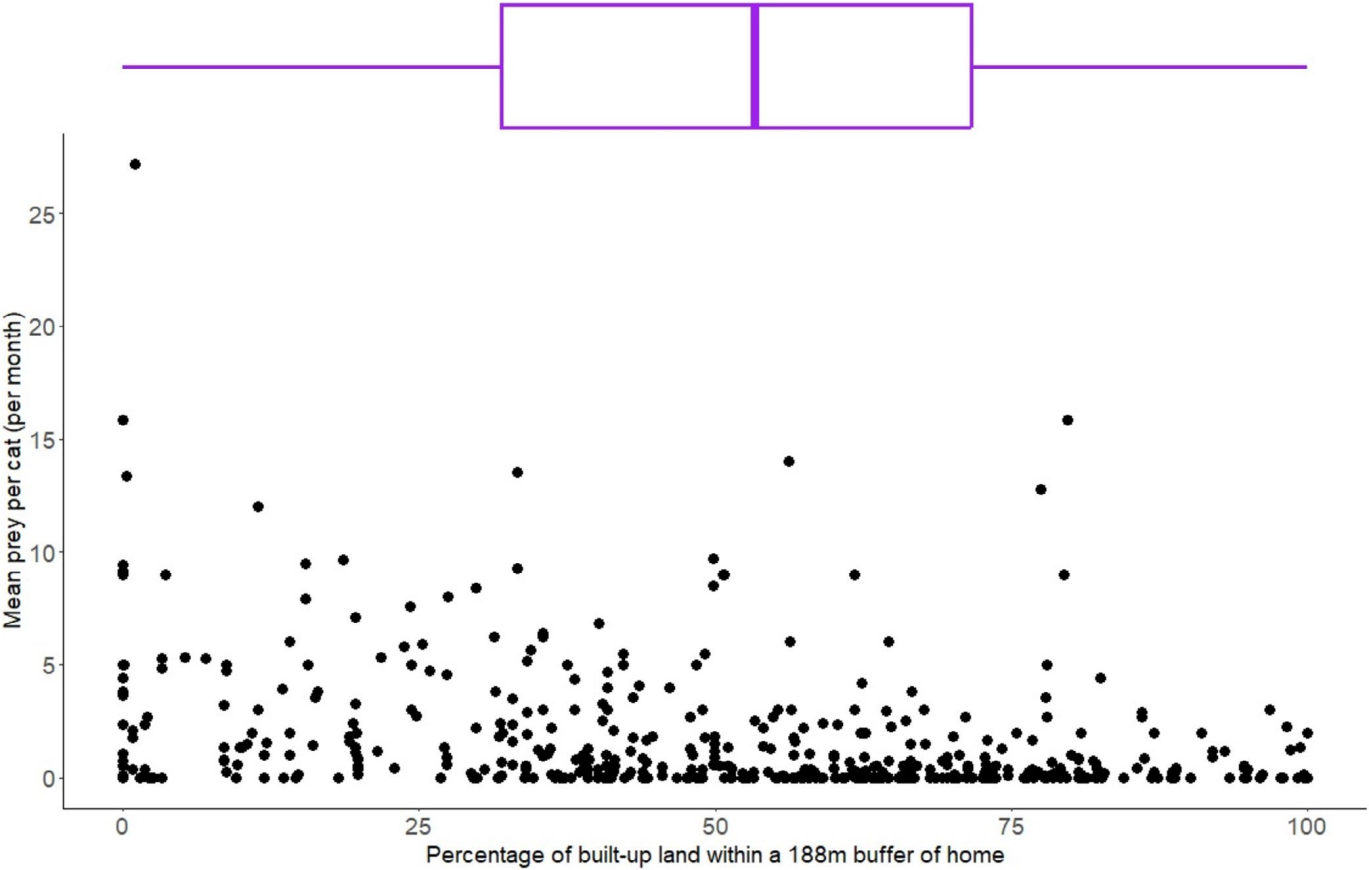
The mean prey returned per month (per cat) against the percentage of built‐up land within a 188 m buffer of home (following the results of Thomas et al. 2012). Each point represents an individual cat and the boxplot above shows the distribution of study cats along this gradient of urbanisation.

There was a statistically important effect of the level of urbanisation within a buffer of the cats' homes, with those in more rural areas returning more prey. However, the upper credible interval in this case was very close to zero (−0.01) and the posterior mean was only −0.02, and therefore it is not an extremely strong effect (Figure 3, Table 2). There was no observed difference between the effect of urbanisation on mammals and birds (Table 2).

An interaction effect was found between most season combinations (except for those between winter and spring, and spring and summer). Overall, bird returns were less affected by seasonal changes than mammal returns, which showed a more pronounced increase in the spring and summer (Table 2). A greater number of prey were returned in the summer months than in any other season, followed by spring (Figure 2, Table 2).

**FIGURE 4:**
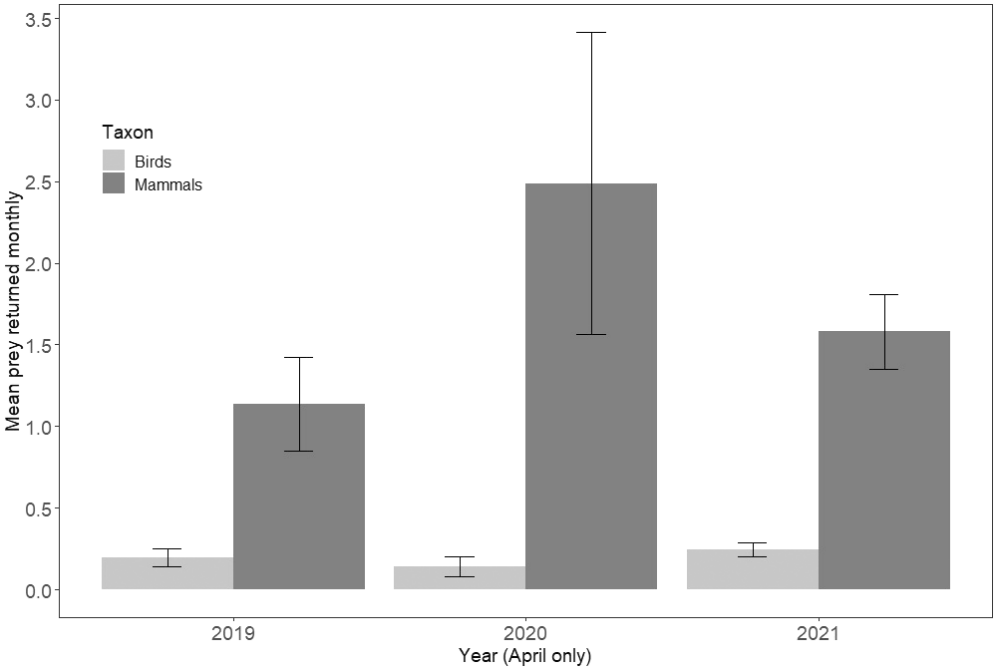
Comparison of mean mammal and bird returns during April 2019, 2020, and 2021, per cat (bars represent Standard Error).

While the mean number of mammals returned per month peaked in 2020, the same year yielded the lowest bird return rates. This was an important interaction between taxon and year. However, aside from this interaction, there were no statistically important findings regarding year (Figure 2, Table 2). On assessment of the raw data, there was a notable increase in prey returns from April 2020 (at the beginning of the COVID‐19 pandemic), which returned to ‘normal’ levels in the autumn of the same year. The mean number of mammals returned for April 2020 was over twice that of the same month in 2019 (2019 mean = 1.14 ± 0.29 SE, 2020 mean = 2.49 ± 0.93 SE), whereas bird returns remained at a similar level (Figure 4).

### 3.2 ‘Super Predator’ Case Studies

The most prolific hunting cat (‘Cally’) returned a total of 362 prey over a 13‐month period, with the vast majority being mammals (98.1%, birds: 1.9%). Cally was a mature adult (aged 7–12 years) female, living in a very rural area in Scotland (only 1.07% built‐up land within the given buffer area). She was of body condition ‘C' (a larger cat, BCS 6–7) and did not have access to a cat‐flap, however she did wear a bell. Another of these super predators named ‘Jet’ returned 97.2% mammals and 2.8% birds. Jet was a mature adult male, of body condition ‘B' (ideal weight, BCS 4–5), who lived in a rural area of England (with only < 1% built‐up land within buffer area). Jet did not wear a belled collar but did have access to a cat‐flap. The third of these prolific hunters, all returning a mean of more than 15 prey per month, was ‘Biggles’, a young adult (aged 1–3 years) male with a body condition of ‘A' (underweight, BCS 1–3). Biggles too had access to a cat‐flap and did not wear a belled collar, but he lived in a more urbanised area (with 79.8% of the buffer zone being built‐up).

### 3.3 Prey Data

The proportion returned alive was highest for amphibians (80.56%, Table 3), followed by reptiles (75%), mammals (20.39%), and birds (14.26%). There were a number of comments in the ‘notes’ section of the data collection forms, stating that individuals were released despite obvious injury. As an example, one stated that a small mammal was ‘just about alive’, suggesting that this individual would not survive for long after release. One bird had ‘lost its tail feathers and [was] unable to fly’, and yet it was released. Of those returned home alive (*n* = 1280), most were released (79.14%) or escaped outdoors (10.31%). A very small number were sent to be cared for at a rescue centre (0.86%), and 2.42% were humanely killed by the cat owner (where injured; for the remaining 7.27%, the fate was unknown).

**TABLE 3:** The number of live and dead prey returned by cats between September 2018 and December 2021, given as total numbers for all taxa.

Taxon	No. dead	No. alive	Total no.
Mammals	4327	1108	5435
Birds	824	137	961
Reptiles	2	6	8
Amphibians	7	29	36

Of the 5835 mammals recorded here, 613 were judged to be either adults or juveniles, by an identification photograph or by the cat owners themselves. Of these, 64.2% were noted as juveniles (Table 4). Similarly, of 505 bird prey items that had been aged, 61.6% were juveniles. Of the 228 brown rats recorded, 106 were aged by participants and of these, 83% were reported to be juveniles (Table 4). Of the 217 rabbit records, 98 had been assigned an age, of which 94.9% were listed as juveniles.

**TABLE 4:** The percentage of prey which were assigned an age category and, of these, the percentage which were identified as juveniles and adults. Key mammalian families are represented here, along with two species. Bird species given represent the five most commonly returned birds. Non‐native species are highlighted with an *.

Prey	Scientific name	Total	% categorised by age	% juvenile	% adult
**All mammals**	**Mammalia**	5835	10.5	64.2	35.8
Murids	Muridae spp.	2865	11.3	73.4	26.6
Voles	Cricetidae spp.	1434	9.6	43.5	56.5
Insectivores	Soricidae spp.	379	12.9	4.1	95.9
Brown rat*	*Rattus norvegicus*	228	46.5	83.0	17.0
European rabbit*	*Oryctolagus cuniculus*	217	45.2	94.9	5.1
**All birds**	**Aves**	1124	44.9	61.6	38.4
House sparrow	*Passer domesticus*	207	36.7	56.6	43.4
Robin	*Erithacus rubecula*	100	54.0	77.8	22.2
Blackbird	*Turdus merula*	102	73.5	53.3	46.7
Blue tit	*Cyanistes caeruleus*	75	56.0	45.2	54.7
Dunnock	*Prunella modularis*	72	50.0	75.0	25.0

### 3.4 Palatability

A total of 379 shrews were recorded throughout the data collection period (6.5% of all mammals, 5.4% of prey overall). The majority (*n* = 310) were returned dead and, of these, 96.8% were reported to be ‘whole and intact’. The remaining 3.2% were noted as being ‘eaten’ (where this was witnessed by the owner) or ‘part‐eaten’ (for example, dismembered or bitten in half, Table 5). This is far lower than for mammals overall (40.4% eaten and part‐eaten).

**TABLE 5:** The percentage of prey which were returned dead and, of these, the percentage that were whole, part‐eaten, or completely eaten (as witnessed by the owner or leaving few remains). The whole, part‐eaten, and eaten percentages do not always total 100%, as some participants did not give these data. Each taxon is represented, and mammals are further divided, giving three key families.

Prey	Total	% returned dead	% whole	% part‐eaten	% eaten
**All mammals**	5835	78.6	59.6	16.3	24.0
Muridae spp.	2865	69.2	68.1	19.9	11.8
Cricetidae spp.	1434	84.1	78.9	15.3	5.6
Soricidae spp.	379	81.8	96.8	2.6	0.3
**All birds**	1124	84.7	66.9	12.3	20.7
**All amphibians**	43	27.9	25.0	58.3	0.0
**All reptiles**	10	25.0	0.0	100.0	0.0

Of the 950 birds returned dead, most were whole (66.9%) and, based on photographs received, appeared to be more easily detectable than mammal remains (S8). In the case of both reptiles and amphibians, the majority were returned alive and most of the dead individuals were either eaten or part‐eaten. In fact, 100% of dead reptiles were found part‐eaten (all of which were slow worms, *Anguis fragilis* , Table 5).

### 3.5 Conservation Concern

Of the 7013 prey returned during the study period, 15 (0.21% of total prey returned) were reported to be European Protected Species: nine bats and six hazel dormice ( *Muscardinus avellanarius* ). However, it should be noted that only one hazel dormouse was confirmed with a photograph, and wood mice ( *Apodemus sylvaticus* ) were misidentified as hazel dormice on three occasions. Mammalian species listed as endangered in the UK (by Mathews and Harrower 2020) were not recorded (such as the water vole, *Arvicola amphibius*, and grey long‐eared bat, *Plecotus austriacus* ). Of the 70 bird species on the UK's red list (Stanbury et al. 2021), five were reported here, totalling 241 individuals (3.44% of total prey returned), with house sparrows ( *Passer domesticus* ) being the most frequently recorded (207 individuals).

However, all of the five prey‐sized mammalian groups commonly perceived as problematic ‘pests’ (mice, brown rats *Rattus norvegicus* , grey squirrels *Sciurus carolinensis* , rabbits *Oryctolagus cuniculus* , and moles *Talpa europaea* , Baker et al. 2020) were recorded here. These groups totalled 3107 individuals (44.4% of total prey returned), potentially more if ‘small mammals’ were assumed to be mice (additional 897 individuals) and if bank and field voles were considered along with mice to be pests (additional 1434 individuals). Three non‐native species were recorded in the present survey, all of which are recognised as pest species (brown rats, grey squirrels, and European rabbits, Baker et al. 2020). These three species constituted 6.5% of total prey returns (*n* = 457 prey).

## 4 Discussion

Here, it was estimated that cats return a mean of 1.5 prey per cat per month, or 18 prey per cat per year. This figure is substantially higher than estimates from recent UK research (5.2 prey per cat per year, Pirie et al. 2022). This difference in estimates may be due to the relatively small geographic range of the study, which was also restricted to urban and suburban environments, and the lower sample monitored by Pirie et al. (2022, *n* = 79). However, the finding of the current study is comparable to that of Woods et al. (2003), estimating that cats in the UK returned 2.04 prey per month (here, it is estimated at 1.5). Since Woods et al. (2003) only monitored prey returns in the spring and summer (5 months), these return rates would likely have been an over‐representation of annual rates. The median produced here (5 prey per cat, annually) is similar to the mean of Pirie et al. (2022), although the median is not routinely given in previous research and is therefore not directly comparable.

A large percentage (44.4%) of prey recorded here is commonly classified as mammalian pests (Baker et al. 2020) and only a very small number (0.21%) were European Protected Species (EPSs). This suggests that in the UK, most prey returned home by cats is of low conservation concern, although it must be recognised that seemingly small losses may be felt more strongly in small, struggling populations (e.g., hazel dormice *Muscardinus avellanarius* , Wembridge et al. 2023). This is not to say that cats do not impact prey populations, but rather that, in the UK, the most commonly caught species are not of high conservation concern like those in Australasia, for example.

### 4.1 Influences on Return Rates

There was an important difference between the predatory habits of male and female cats, suggesting that males return a greater number of prey than females. Although the majority of research indicates that there is no discernible difference in predation between sexes (Barratt 1998; Gillies and Clout 2003; Morgan et al. 2009; Tschanz et al. 2011; Loyd et al. 2013; Kauhala et al. 2015), the sample sizes tested here were relatively large and balanced (males *n* = 2566 data months from 284 cats, females *n* = 2108 data months from 269 cats), which improves the reliability of such a result. This finding may be linked to the range size and roaming distance of male and female cats. As males are known to have larger ranges (Liberg et al. 2000), it may be likely that their roaming areas contain a greater number of small mammal burrows, for example, therefore providing them with greater hunting opportunities.

The age of the cats in this study also proved to be an important variable, influencing prey return rates. The kitten age group (aged < 1 year) had the highest monthly mean prey return value (from the raw data), although this was not apparent in the Bayesian GLMM test output. This is likely due to the relatively low sample size for this age group (*n* = 11 cats, *n* = 71 data months). While a balanced sample would be beneficial here, the inclusion of a large number of kittens would likely not be representative of the wider population. Generally, as cats increased in age, prey returns reduced for both mammals and birds. A similar result has been found previously (Barratt 1998; Howes 2002; Flux 2007; Morgan et al. 2009; van Heezik et al. 2010; Bruce et al. 2019), although some have found no pattern between cat age and prey return rates (in metropolitan Australia, Robertson 1998; and an isolated Swiss town, Tschanz et al. 2011). While one of these studies used a relatively small sample of cats (*n* = 32, Tschanz et al. 2011), the other was based on owner recollection and simply whether each cat had returned prey in the previous 12 months (i.e., the number of prey returned was not tested, Robertson 1998). Given a larger sample size, it is possible that variables (including age) which were not found to be of importance may become significant. Furthermore, the methods used by Robertson (1998, i.e., simply looking at predators and non‐predators) are rather different from the majority which assess the number of prey returned. Therefore, the results of this study (Robertson 1998) are not directly comparable to most others and less powerful at detecting differences.

The Body Condition Score of an individual is likely related to its fed diet (Silva‐Rodriguez and Sieving 2011), although it can be a result of illness (Bellows et al. 2016), neutering status (Scott et al. 2002), or age (Crawford et al. 2020). However, body condition may be related to predation in a number of ways. It could be assumed that, if cats are indeed supplementing their diet with wild prey, they are perhaps less likely to be particularly thin (Silva‐Rodriguez and Sieving 2011). However, cats which are fed less at home (having a lower BCS) may have a greater motivation to hunt, and therefore return a greater number of prey. Biben (1979) found that the probability of a cat killing its prey is reduced for those which are well‐fed. Similarly, Cecchetti et al. (2021) found that a diet high in meat protein can reduce the number of prey returned. However, there is evidence to suggest that cats will hunt regardless of hunger (at least to some extent, Adamec 1976; Leyhausen 1979; Turner and Bateson 2000). It is probable that leaner cats may be more agile and able to hunt with greater success, although research is lacking in this area. Furthermore, all of the ‘kitten’ age cats monitored here were registered as being of either ‘A' or ‘B' body condition (thinner and leaner). It is likely that as these cats develop into adults, some will become larger (22.1% of young adults were in category ‘C' or ‘D'). Therefore, age and body condition effects may be linked. Indeed, Smit et al. (2022) found that with age, body condition score increased, as physical activity decreased.

Although not an ‘important’ result, generally the presence of a cat flap led to greater prey return figures, which may be simply due to an increase in detection rates. For example, prey items left outside the house may be scavenged or otherwise go unnoticed and unrecorded, whereas the use of a cat flap means that cats can more easily return prey indoors, therefore increasing the number observed by owners. It is also likely that the implementation of a cat flap will influence the total time a cat spends outdoors. However, it should be noted that this is largely speculative, as cat activity budgets and cat flap access have not previously been evaluated. Cat flaps may also influence the time of day that cats are outdoors, such that those without cat flaps will likely be either locked inside or outside for the duration of the night, whereas those with cat flap access are able to roam freely. This may have an effect on the proportions of different prey taxa returned, as activity times can vary by taxonomic grouping (e.g., all birds caught here were diurnal and most rodents, nocturnal). Although both mammals and birds followed a similar pattern in that returns were higher for both when a cat flap was present, having unlimited access to and from the outdoors (via a cat flap) affected returns of mammalian prey more strongly than that of birds. Although this may be linked with the time of day that cats with or without cat flap access are able to roam outdoors (with increased mammalian activity between dusk and dawn), it may also be due to the higher detectability of birds (whether outdoors or indoors). This is because feathers are often more visible and less likely to be overlooked than mammal remains (e.g., a tail; S8). This variable has not been investigated in previous literature, despite its clear influence.

The increased return rates of cats wearing bells are in contrast to many previous studies (Ruxton et al. 2002; Nelson et al. 2005; Gordon et al. 2010), which have consistently found a reduction in both mammal and bird returns, although Morgan et al. (2009) observed no significant effect and Woods et al. (2003) found a reduction in mammalian prey, but not in birds. The studies which found that bells reduce all prey returns had a paired design, where the same sample of cats were monitored with and without a bell (Ruxton et al. 2002; Nelson et al. 2005; Gordon et al. 2010). Therefore, those which did not usually wear a belled collar (all cats in the case of Ruxton et al. 2002) were not used to it and certainly not used to stalking prey successfully with its presence. However, studies which take a cross‐sectional approach compare cats which usually wear bells to cats that do not. Additionally, it may be that the already prolific hunters are fitted with belled collars in an attempt to limit predation, whereas those that rarely return prey are left without collars, or at least without bells attached. Few cross‐sectional prey return surveys investigate the effect of a belled collar, perhaps because there are many variables which cannot be controlled in such a study (e.g., fed diet, location, prey availability, season, and age). Here, it was not possible to include interaction terms within the model, due to computational power limits. However, it would be beneficial to explore the effect of bell use on predation rates, depending on intrinsic variables, such as age.

Although here, there was a statistically important relationship between urbanisation level (indicated by the proportion of built‐up land within a ranging buffer) and prey return figures, this was a weak relationship and ‘super predator’ cats were observed in even highly urban areas. However, the general trend may be explained by range size, since cats in more rural areas (living in lower densities) have been observed to travel greater distances (Churcher and Lawton 1987; van Heezik et al. 2010), likely having a greater prey population within their range. While the current study found no taxon interaction, previous research has found a rather common pattern, as in Poland (Krauze‐Gryz et al. 2017), Finland (Kauhala et al. 2015), and Australia (Barratt 1997), bird return rates increased as the environment became more urban. The results presented here show a rather even spread of cats living in areas across this gradient of urbanisation and, therefore, if there were any patterns like these in the UK, they would likely have been observed. Although not monitored here, due to overparameterisation concerns, given a larger sample size, the generalist or specialist nature of prey could be looked into across an urbanisation gradient. Since it is clear from previous research that cats capture prey according to availability (Churcher and Lawton 1987; Molsher et al. 1999; Loyd et al. 2013; Krauze‐Gryz et al. 2017), predation studies such as the one presented here could potentially be used as a tool to monitor rodent populations, for example. Monitoring mortality rates as a proxy for population size and health has been shown to be an effective method (e.g., recording road‐killed animals, Bíl et al. 2020).

For both mammals and birds, prey returns were highest in the summer months (June–August), followed by spring (March–May), autumn (September–November), and winter (December–February). While some previous research on feral cats in Australia has found no evidence of seasonal changes in predation (Hutchings 2003), a number of studies (also on feral cats in Australia) have identified significant patterns, with diet containing a higher quantity of small‐bodied prey (such as invertebrates) when larger species are unavailable (Molsher et al. 1999; Kutt 2011; Woinarski et al. 2018). A similar seasonal pattern has also been found in pet cats in the UK (Howes 2002). It may be expected that predation would follow the breeding patterns of prey species, as cats are known to predate in accordance with prey availability (Churcher and Lawton 1987; Molsher et al. 1999; Loyd et al. 2013; Kitts‐Morgan et al. 2015). These seasonal variations may therefore be explained by the annual activity patterns of prey and times of the year when they may be more vulnerable to predation (e.g., when foraging behaviour is highest).

An increase in prey returns was observed from April 2020 (at the beginning of the COVID‐19 pandemic). A study of human activity and bird presence records shows that when people were at home during ‘lockdown’ periods, sightings of most species declined (compared to pre‐COVID‐19 levels, Warrington et al. 2022). There were also reports of mammals (particularly larger‐bodied species such as deer and wild boar) roaming towns and cities during this time, as human disturbance was severely reduced (as reviewed by Miraglia and Di Brita 2022). Although not reviewed, it is perhaps likely that small mammal species followed the same pattern as birds, reducing their suburban and garden activity as people spent more time in these environments. However, the results presented here suggest an increase in either prey returned by cats or those detected by owners. It is likely that National lockdowns increased cat owners' time at home and therefore either increased detection throughout the day or allowed those cats which would usually be confined to the house during the day (with no cat flap) to go outdoors and catch a greater number of prey.

One might expect that the top predators are of a young age with a low BCS, according to the results presented here. However, of the three ‘super predator’ cats included (those returning an average of ≥ 15 prey per month), just one matched this profile (‘Biggles’). Indeed, the top predator, ‘Cally’, was a larger‐bodied, mature adult female, although she lived in a very rural location. With only three cats returning these high monthly figures, the only reasonable interpretation of these data is that there is high variability between individuals and there are likely many factors affecting predatory habits which were not accounted for here (such as dietary factors and personality markers).

### 4.2 Prey Returns

Of those prey discovered alive, around 80% were released, seemingly regardless of injuries sustained. This perhaps highlights an avenue of further study, investigating the drivers behind cat owners' attitudes and actions regarding returned prey. While prey age data should be cautiously interpreted (due to only confident owners giving this extra information), a similar proportion of mammals and birds were identified as being juveniles (64.17% and 61.58%, respectively). This may be a reflection of the availability of prey throughout the study period. A five‐year study into the demographics of a British passerine (bullfinch, *Pyrrhula pyrrhula* ) found that at its peak (October), the population contained over three times as many juveniles as adults (using mist nets, Newton 1999). This study suggests that juveniles can make up around 78.5% of the total population (in the Autumn, Newton 1999), and therefore, the results found here add further evidence to the suggestion that cats hunt in accordance with what is available (Churcher and Lawton 1987; Molsher et al. 1999; Loyd et al. 2013; Kitts‐Morgan et al. 2015; Yip et al. 2015).

However, as juvenile birds are generally dimorphic from adults, at least until their post‐juvenile moult, and are therefore quite easy to identify as such, age may have been more frequently noted for young individuals. Age was not explicitly requested on data entry forms, and therefore, many participants may have assumed that this information was not useful (or were not certain). This could have led to many people only recording age when reporting an obvious juvenile. As a result, young birds (and mammals) may be overrepresented in the data, and it would therefore be incorrect to assume that the entire dataset follows these proportions.

Of the mammal returns, it was apparent throughout data collation that many brown rats and European rabbits were reported to be juveniles, so this was examined further. The high proportion of brown rat juveniles may be considered representative, as adults are known to be very aggressive and are therefore less likely to be caught (Fitzgerald et al. 1991). Brown rats can also be relatively easily aged by size, which may have increased participants' ability to accurately collect these data. Of the European rabbits reported, just under half (45.16%) were assigned an age, largely because many were not returned whole (and therefore could not be easily aged). The high number of juveniles in this sample appears to represent availability, as a large increase in rabbit records was observed in spring and summer months (when juveniles are plentiful). Indeed, Flux (2007) found in New Zealand that 97.14% of European rabbits returned home were juveniles (in New Zealand), comparable to the 94.9% found here. However, larger prey (such as adult rabbits) may be less likely to be returned home, perhaps also dependent on the distance and habitat between the capture site and the house.

### 4.3 Importance of Palatability

Another factor recorded regarding the prey returned was whether the individual was eaten, part‐eaten, or left whole by the cat. Here, the high proportion of shrews observed being returned ‘whole and intact’ (96.8%) provides further evidence to suggest that insectivores, or at least shrew species, are generally unpalatable to cats (as suggested by Toner 1956; Leyhausen 1979; Turner and Bateson 2000; Krauze‐Gryz et al. 2012; Kauhala et al. 2015). This may lead to a higher proportion of total predated shrews (total captures) being recorded in return‐questionnaire studies, than other (more palatable and thus less likely detected) small mammals. This strongly suggests that it would be inappropriate to extrapolate these data (in order to reach a figure for total predation) using the same multiplication factors as for more palatable taxa. For example, where Kays and DeWan (2004) estimated that cats return 30% of prey killed, all returned taxa were multiplied by 3.3. Here, the data suggest that, for insectivores, this multiplication factor should be far lower than that of rodents for example, as rodents are eaten in much higher proportions. Similarly, as birds are perhaps more detectable than small mammals (see S8), these prey should also be multiplied by a lower figure than rodents.

There may also be differences in the palatability and detectability of amphibians, reptiles, and invertebrates, although the data retrieved here for these taxa were not substantial enough to draw any conclusions. This type of analysis would be better conducted on data from warmer climates, where large volumes of herptiles are returned home. The results of questionnaire studies may always be subject to biases, due to differences in the detectability of remains and the palatability of different taxa.

### 4.4 Limitations

Factors which affect or influence hunting behaviour in cats are likely complex and are not all measurable in a large‐scale study. For example, the personality traits of each cat (along with those of cohabiting individuals), attention received at home and the nature of attention or play, an in‐depth look at home diet and feeding regime, the cats' history (early life, adoption, environment, health), and fine‐scale assessment of the surrounding habitat are not easily obtained. While Cecchetti et al. (2021) investigated many factors including diet and play time, this was not a cross‐sectional study of already established routines but included instruction for the introduction of play time, for example (paired design). Although such research programmes are highly valuable, they do not always account for ingrained routines and behaviours. The approach of the current study was to monitor the basic attributes of cats, along with existing husbandry practices and routines, in order to assess the wider population rather than to alter practices in an experimental way. It is important that the extant situation is measured.

It is assumed here that the study population is representative of cats across the UK. Although there is no complete ‘cat census’ including all of this information on a national scale, with a large enough sample size and no reason to suggest biases in recruitment, the sample presented here should be very close to that of the wider population. Nonetheless, this is a large assumption, and despite efforts made to create an unbiased dataset, the results should be interpreted with this in mind.

The reliability of data presented here could be improved in future if a higher proportion of prey return data include one or more photographs for identification by researchers. However, only 7.12% of photographed prey were misidentified by cat owners and, regarding those of high conservation concern, it was common species (such as wood mice) which were occasionally reported as those of concern (e.g., hazel dormice). This suggests that the estimate of species of concern was more likely an overestimate than an underestimate. Since this study was long‐term with ongoing recruitment of participants throughout, it would not be possible to provide more formal identification training, and thus, photographic confirmation is of high importance.

Although the sample size of cats and data months were substantial here, only one interaction effect (between taxon and all other variables) was measured. A greater number of interaction terms and categories within variables would have created overparameterisation of the data. However, given a much larger, balanced cat sample, the inclusion of both more interactions in the model and a greater number of categories within each variable (e.g., age categories) would be possible.

Additionally, the extrapolated figures presented here (37.25 million–140.4 million prey per year) were generated using two distinct methods: the median of 194 cats and the mean of 553 cats, respectively. The estimate based on the median represents the effects that the vast majority of cats have on the prey populations, while the estimate based on the mean highlights the impact of a small proportion of super predators (1.4% of the total sample monitored here returned ten or more items per month). This also suggests that if it is aimed to limit the impact of cats on specific prey species of concern, efforts should be focused on super predators, unless they actually predate on problematic species such as brown rats, or cats that live close to a population of a prey species of concern and are known to prey on these. If the predation rates of super predator cats can be reduced to the level of the average cat, the overall predation pressure could be reduced to about a quarter of the current level. This also highlights that caution must be exercised when interpreting these calculations, particularly if using them in further study, to avoid under‐ or over‐estimation of predation by cats.

## 5 Conclusions

Here, the influence of eight key factors was tested, and while some (age, sex, year, and season) cannot be controlled by cat owners, there are a few things that owners could do which may reduce predation. Body condition may be controlled in some individuals (although this can depend on overall health and metabolism) by altering the type and quantity of food provision. The removal (or locking) of an existing cat flap may also reduce predation at certain times of the day, allowing owners to control when their cat has access to the outdoors. However, it is important to note that locking cats indoors overnight and allowing them outdoor access during the day could increase the relative proportion of birds to mammals preyed on, at least in the UK (Lockwood 2024).

It was noted here that only a very small proportion of shrews (3.2%) were reported as being eaten or part‐eaten, which adds further evidence supporting the idea that shrews are unpalatable to cats. Therefore, insectivore species should certainly be treated differently when extrapolating to produce a figure of total predation by cats (which includes those unrecorded in questionnaire‐based studies). This also highlights the need to take detectability and palatability into account when trying to predict predation from return rates.

Although broad estimates of annual prey returns were presented here, return rates are likely lower than actual predation rates (as found in video studies e.g., Loyd et al. 2013; Seymour et al. 2020). Since the estimates of the proportion of total catch returned home in direct observation studies vary from 8.8% (estimated by comparing consumed and returned prey in Poland, Krauze‐Gryz et al. 2012) to 30% (estimated based on direct observation in the USA, Kays and DeWan 2004), further investigation into return rates for prey in the UK is warranted. It is important to recognise that neither return rates nor total predation rates allow a direct inference on whether prey populations are affected by cats, and therefore further research on impact is necessary (Baker et al. 2005, 2008).

## Author Contributions

**H. L. Lockwood:** data curation (lead), formal analysis (lead), methodology (lead), project administration (lead), writing – original draft (lead), writing – review and editing (lead). **M. Bulling:** formal analysis (supporting), writing – review and editing (supporting). **M. Huck:** formal analysis (supporting), supervision (supporting), writing – original draft (supporting), writing – review and editing (supporting).

## Conflicts of Interest

The authors declare no conflicts of interest.

## Supporting information


Data S1.

## Data Availability

Data are stored via Dryad (https://datadryad.org/stash/share/V8oXOtdGhv9Mai9hx8jweqi7buWR6oTLCwglTCqvO7I).
