# Supplementary material for: What the Cat Dragged in: Quantifying Prey Return Rates of Pet Cats (Felis catus) With Outdoor Access in the UK
Source: Ecol Evol. 2025 Mar 6;15(3):e71063. doi: 10.1002/ece3.71063 (PMC11884926; doi:10.1002/ece3.71063)
Supplement: Supplementary file 1 — Data S1. [file ECE3-15-e71063-s001.docx]

Supporting information

**S1.** The questions asked in the cat registration survey.

| **Question no.** | **Question** |
| --- | --- |
| 1 | Your full name |
| 2 | Your postcode |
| 3 | Your email address |
| 4 | How many cats live in your household? |
| 5 | Your cat’s name |
| 6 | How old is your cat? |
| 7 | Please tell us the breed or colouration of your cat |
| 8 | Sex of cat |
| 9 | Has your cat been neutered? |
| 10 | IF YES: At what age was your cat neutered? |
| 11 | Using the diagram below, how would you categorise your cat's body shape?  (See S2) |
| 12 | What food do you provide for your cat? |
| 13 | Does your cat have access to a cat-flap? |
| 14 | Does your cat wear a collar with a bell on it? |
| 15 | Do you regularly provide food for wild birds in your garden? |
| 16a | On average, how many dead small mammals or birds does your cat bring home per month? April- September |
| 16b | On average, how many dead small mammals or birds does your cat bring home per month? October- March |
| 17a | On average, how many living small mammals or birds does your cat bring home per month? April- September |
| 17b | On average, how many living small mammals or birds does your cat bring home per month? October- March |
| 18a | On average, how many hours per day does your cat spend outside? April- September |
| 18b | On average, how many hours per day does your cat spend outside? October- March |
| 19 | Please use the space below to tell us about any current health problems which you think may influence your cat's behaviour. |
| 20 | Are you interested in taking part in our GPS and/or cat-camera study? |


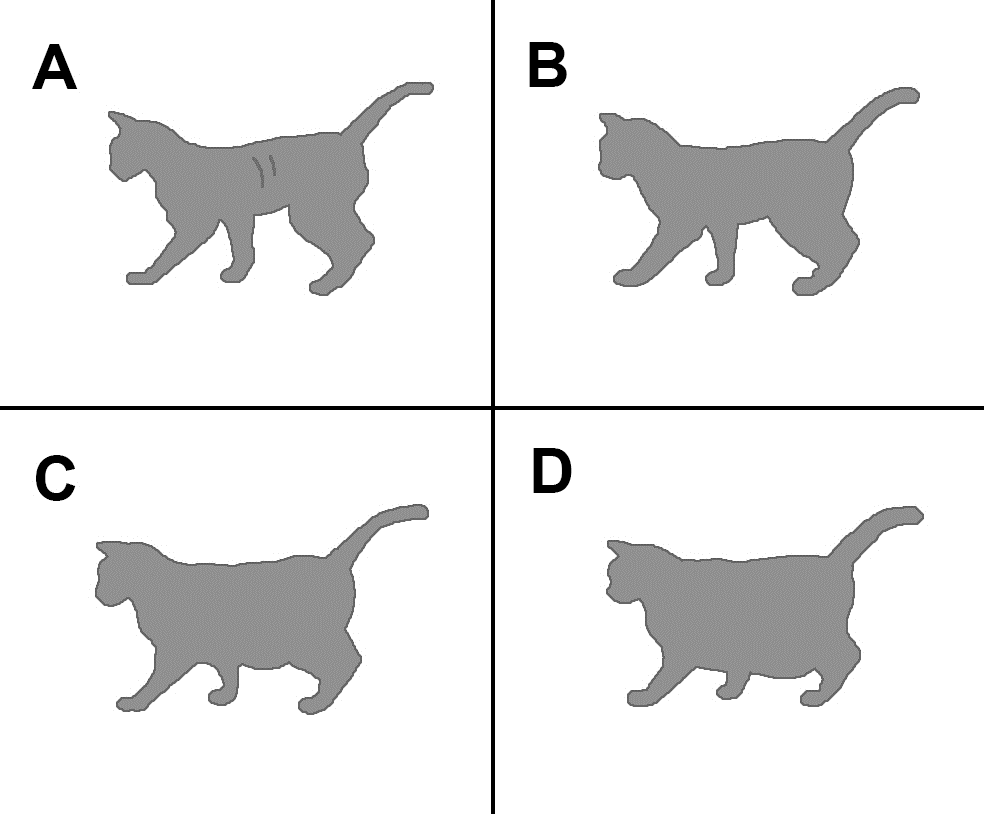


**S2.** Body Condition Score (BCS) visual categories (A, B, C, and D), selected by each cat owner during the registration process. ‘A’ represents underweight cats (BCS of 1-3), ‘B’ represents ideal condition (BCS of 4-5), ‘C’ represents overweight individuals (BCS of 6-7), and ‘D’ represents obese cats (BCS of 8-9). Image adapted from Purina (n.d.).


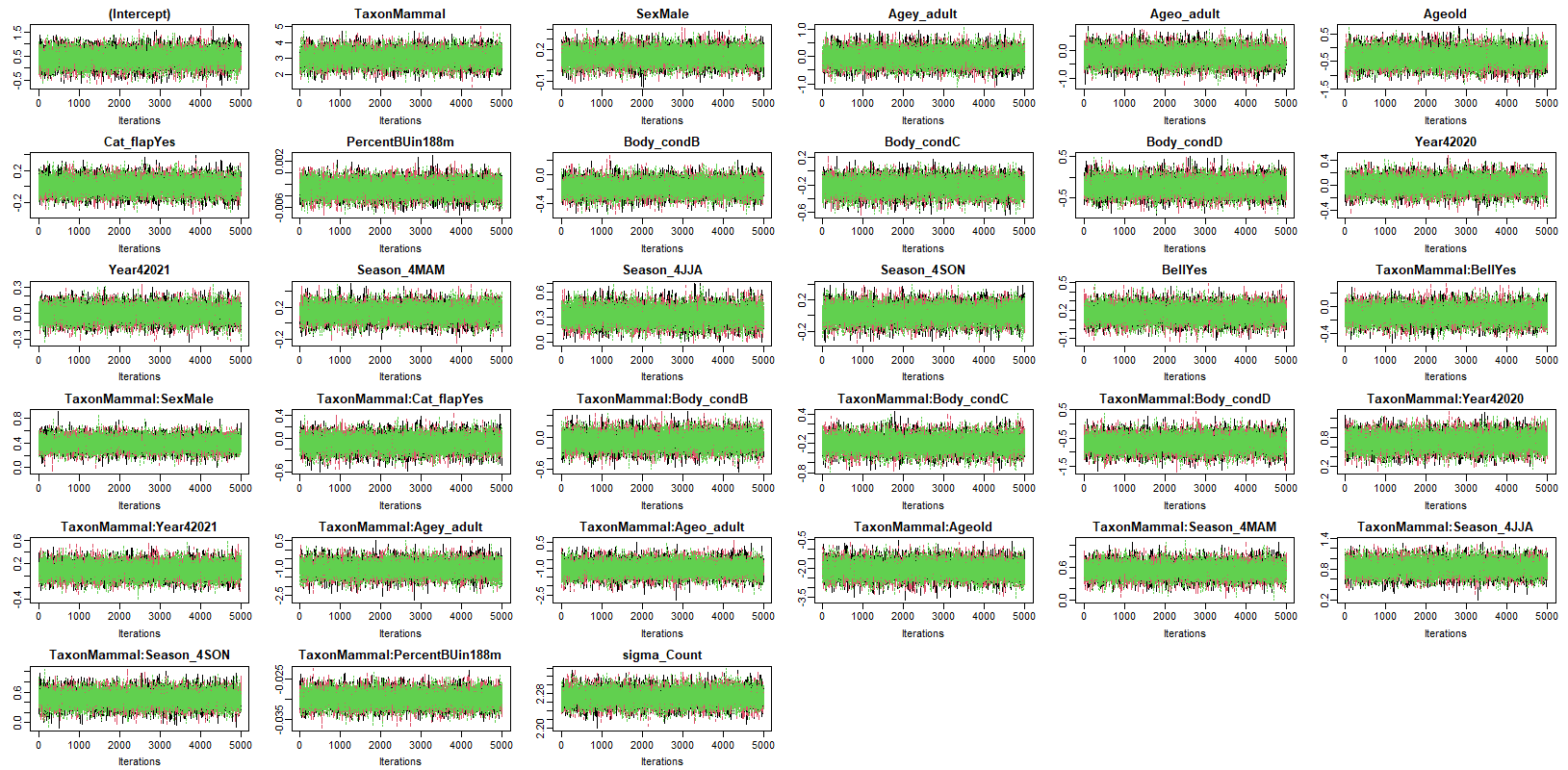


**S3.** Trace plots showing the convergence of chains (or ‘mixing’), used in the final model.

**S4.** All prey return records, by species. Where a broader taxonomic group is given (e.g. Muridae sp.), identification to species level was not achieved. Totals for each taxon are given in bold, following the species names, as is a total of all prey recorded. Common names of non-native species are underlined.

| **Class** | **Latin name** | **Common name** | **Total** | **% of all prey** |
| --- | --- | --- | --- | --- |
| Mammalia |  | Small mammal | 897 | 12.79 |
|  | Muridae sp. | (Mouse) | 874 | 12.46 |
|  | *Apodemus sylvaticus* | Wood mouse | 1446 | 20.62 |
|  | *Apodemus flavicollis* | Yellow-necked mouse | 27 | 0.39 |
|  | *Mus musculus* | House mouse | 274 | 3.91 |
|  | *Micromys minutus* | Harvest mouse | 16 | 0.23 |
|  | *Rattus norvegicus* | Brown rat | 228 | 3.25 |
|  | Cricetidae sp. | (Vole) | 203 | 2.90 |
|  | *Myodes glareolus* | Bank vole | 551 | 7.86 |
|  | *Microtus agrestis* | Field vole | 680 | 9.70 |
|  | *Muscardinus avellanarius* | Hazel dormouse | 6 | 0.09 |
|  | *Sciurus carolinensis* | Grey squirrel | 12 | 0.17 |
|  | *Talpa europaea* | European mole | 13 | 0.19 |
|  | Soricidae sp. | (shrew) | 125 | 1.78 |
|  | *Sorex araneus* | Common shrew | 141 | 2.01 |
|  | *Sorex minutus* | Pygmy shrew | 107 | 1.53 |
|  | *Neomys fodiens* | Water shrew | 6 | 0.09 |
|  | Chiroptera sp. | (Bat) | 4 | 0.06 |
|  | *Pipistrellus sp.* | (Pipistrelle bat) | 3 | 0.04 |
|  | *Plecotus auritus* | Brown long-eared bat | 1 | 0.01 |
|  | *Myotis nattereri* | Natterer's bat | 1 | 0.01 |
|  | *Oryctolagus cuniculus* | European rabbit | 217 | 3.09 |
|  | *Erinaceus europaeus* | Hedgehog | 1 | 0.01 |
|  | *Mustela erminea* | Stoat | 1 | 0.01 |
|  | *Mustela nivalis* | Weasel | 1 | 0.01 |
|  |  | **Mammals** | **5835** | **83.21** |
| Aves |  | Bird | 187 | 2.67 |
|  | Paridae sp. | (Tit) | 6 | 0.09 |
|  | *Cyanistes caeruleus* | Blue tit | 75 | 1.07 |
|  | *Parus major* | Great tit | 29 | 0.41 |
|  | *Aegithalos caudatus* | Long-tailed tit | 9 | 0.13 |
|  | *Poecile palustris* | Marsh tit | 1 | 0.01 |
|  | *Periparus ater* | Coal tit | 4 | 0.06 |
|  | *Prunella modularis* | Dunnock | 72 | 1.03 |
|  | *Troglodytes troglodytes* | Wren | 66 | 0.94 |
|  | *Sturnus vulgaris* | Starling | 17 | 0.24 |
|  | Turdidae sp. | (Thrush) | 6 | 0.09 |
|  | *Turdus merula* | Blackbird | 102 | 1.45 |
|  | *Turdus iliacus* | Redwing | 1 | 0.01 |
|  | *Turdus philomelos* | Song thrush | 4 | 0.06 |
|  | *Erithacus rubecula* | Robin | 100 | 1.43 |
|  | *Sylvia atricapilla* | Black cap | 4 | 0.06 |
|  | *Regulus regulus* | Goldcrest | 3 | 0.04 |
|  | *Passer domesticus* | House sparrow | 207 | 2.95 |
|  | Fringillidae sp. | (Finch) | 5 | 0.07 |
|  | *Chloris chloris* | Greenfinch | 14 | 0.20 |
|  | *Fringilla coelebs* | Chaffinch | 25 | 0.36 |
|  | *Carduelis carduelis* | Goldfinch | 35 | 0.50 |
|  | *Pyrrhula pyrrhula* | Bullfinch | 7 | 0.10 |
|  | *Linaria cannabina* | Linnet | 1 | 0.01 |
|  | *Carduelis spinus* | Siskin | 2 | 0.03 |
|  | Columbidae sp. | (Pigeon/dove) | 31 | 0.44 |
|  | *Streptopelia decaocto* | Collared dove | 9 | 0.13 |
|  | *Columba palumbus* | Wood pigeon | 58 | 0.83 |
|  | *Columba livia* | Feral pigeon | 17 | 0.24 |
|  | Corvidae sp. | (Corvid) | 1 | 0.01 |
|  | *Corvus monedula* | Jackdaw | 2 | 0.03 |
|  | *Pica pica* | Magpie | 4 | 0.06 |
|  | *Dendrocopos major* | Great spotted woodpecker | 4 | 0.06 |
|  | *Sitta europaea* | Nuthatch | 2 | 0.03 |
|  | *Certhia familiaris* | Treecreeper | 3 | 0.04 |
|  | *Delichon urbicum* | House martin | 2 | 0.03 |
|  | *Apus apus* | Swift | 1 | 0.01 |
|  | *Motacilla cinerea* | Grey wagtail | 1 | 0.01 |
|  | *Psittacula krameri* | Ring-necked parakeet | 2 | 0.03 |
|  | Laridae sp. | (Gull) | 1 | 0.01 |
|  | Anatidae sp. | (Duck) | 1 | 0.01 |
|  | *Anas platyrhynchos* | Mallard | 3 | 0.04 |
|  |  | **Birds** | **1124** | **16.03** |
| Reptilia | *Anguis fragilis* | Slow worm | 6 | 0.09 |
|  | *Zootoca vivipara* | Common lizard | 4 | 0.06 |
|  |  | **Reptiles** | **10** | **0.14** |
| Amphibia | *Rana temporaria* | Common frog | 38 | 0.53 |
|  | *Pelophylax ridibundus* | Marsh frog | 1 | 0.01 |
|  | Bufonidae sp. | (Toad) | 2 | 0.03 |
|  | *Bufo bufo* | Common toad | 3 | 0.04 |
|  |  | **Amphibians** | **44** | **0.61** |
|  |  | **Total prey** | **7013** |  |


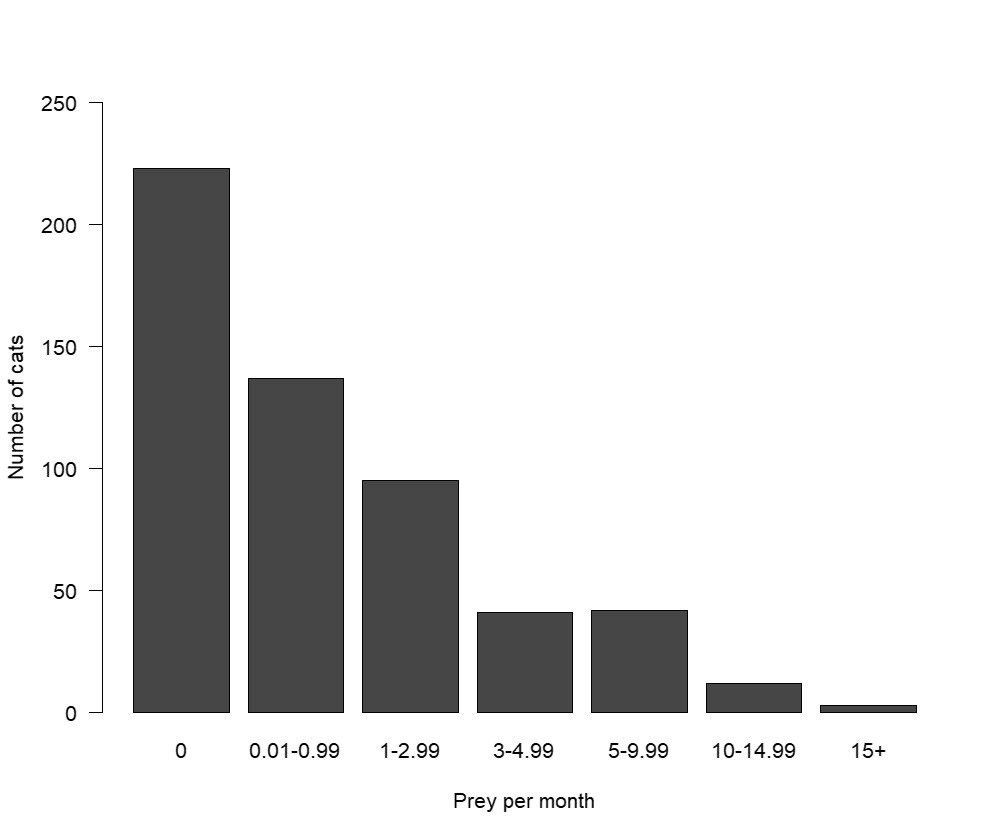


**S5.** The mean number of prey animals returned each month, and the number of cats returning each category.

**S6.** Numerical outputs of Bayesian negative binomial GLMM, testing for inclusion or exclusion in the final model. Posterior mean (P mean), standard error (SE), and the 2.5% and 97.5% credible intervals are given. Where credible intervals do not cross zero, a statistically important result is observed. Results in bold show important differences, and those bold variables were selected for inclusion in the final model.

| **Variable** | **P mean** | **SE** | **2.5%** | **97.5%** |
| --- | --- | --- | --- | --- |
| Taxon: Birds < Mammals | **1.3** | **0.34** | **0.68** | **2.03** |
| Sex: Female < Male | **0.5** | **0.19** | **0.13** | **0.82** |
| Age: Kitten < Young adult | **0.7** | **0.23** | **0.33** | **1.16** |
| Kitten > Old adult | **-0.5** | **0.24** | **-0.90** | **-0.01** |
| Kitten > Old | **-1.8** | **0.41** | **-2.44** | **-1.15** |
| Body condition: A > B | **-0.6** | **0.26** | **-1.03** | **-0.07** |
| A > C | **-1.3** | **0.25** | **-1.81** | **-0.74** |
| A > D | **-1.5** | **0.53** | **-2.19** | **-0.48** |
| Belled collar: No < Yes | **0.5** | **0.21** | **0.12** | **0.97** |
| Season: Winter < Spring | **0.7** | **0.16** | **0.43** | **0.98** |
| Winter < Summer | **1.3** | **0.18** | **1.00** | **1.58** |
| Winter = Autumn | -0.02 | 0.20 | -0.37 | 0.35 |
| Year: 2019 > 2020 | **-0.2** | **0.11** | **-0.42** | **-0.03** |
| 2019 = 2021 | -0.1 | 0.15 | -0.30 | 0.24 |
| Neutered: No = Yes | 0.2 | 0.66 | -0.53 | 1.97 |
| Cat flap: No < Yes | **0.7** | **0.18** | **0.34** | **1.03** |
| Percentage Built-up (buffer) | **-0.03** | **0.004** | **-0.04** | **-0.02** |

**S7.** Number of cats in each specified category, along with the number of ‘data months’, and total number of mammals and birds returned. Data displayed are adjusted (rounded up or down), as detailed in the ‘data preparation’ section of the main manuscript. Number of cats in the age categories do not total 553 (actual number of individuals), as some changed category over the study period.

| **Category** | **No. of cats** | **Data months** | **Prey: mammals** | **Prey: birds** |
| --- | --- | --- | --- | --- |
| **Sex** |  |  |  |  |
| Male | 284 | 2566 | 3913 | 753 |
| Female | 269 | 2108 | 2194 | 408 |
| **Age** |  |  |  |  |
| Kitten | 14 | 95 | 216 | 45 |
| Young adult | 312 | 2319 | 3447 | 821 |
| Mature adult | 182 | 1742 | 2336 | 278 |
| Senior | 53 | 518 | 108 | 17 |
| **Body condition** |  |  |  |  |
| A | 104 | 790 | 1375 | 321 |
| B | 291 | 2334 | 2996 | 573 |
| C | 141 | 1349 | 1624 | 243 |
| D | 17 | 201 | 112 | 24 |
| **Bell?** |  |  |  |  |
| Yes | 147 | 1252 | 1605 | 435 |
| No | 406 | 3422 | 4502 | 726 |
| **Neutered?** |  |  |  |  |
| Yes | 541 | 4584 | 6063 | 1152 |
| No | 12 | 90 | 44 | 9 |
| **Cat flap?** |  |  |  |  |
| Yes | 413 | 3703 | 5102 | 971 |
| No | 140 | 971 | 1005 | 190 |
| **Season** |  |  |  |  |
| Winter | 408 | 1177 | 779 | 137 |
| Spring | 374 | 1096 | 1540 | 278 |
| Summer | 390 | 1156 | 2124 | 549 |
| Autumn | 426 | 1245 | 1664 | 197 |
| **Year** |  |  |  |  |
| 2019 | 162 | 988 | 1120 | 255 |
| 2020 | 223 | 655 | 999 | 107 |
| 2021 | 305 | 2330 | 3069 | 554 |


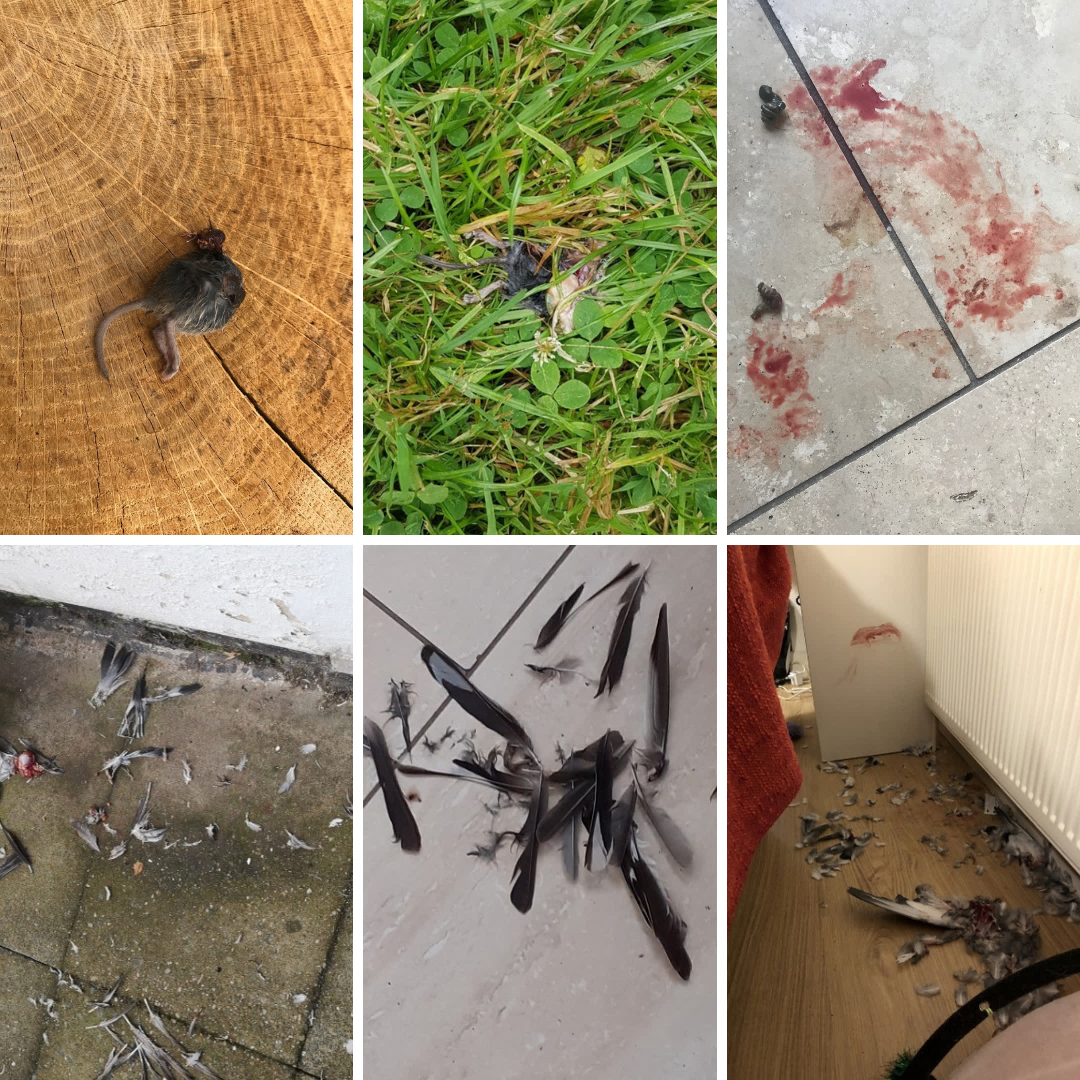


**S8.** Comparison of a typical sample of small mammal remains (top) and bird remains (bottom).
